# Physiotherapy services in patient care in Bhutan

**DOI:** 10.1186/s12960-021-00649-1

**Published:** 2021-09-03

**Authors:** Monu Tamang, Thinley Dorji

**Affiliations:** 1 Physiotherapy Unit, Central Regional Referral Hospital, 31101 Gelegphu, Bhutan; 2 Department of Internal Medicine, Jigme Dorji Wangchuck National Referral Hospital, Thimphu, Bhutan; 3 Kidu Mobile Medical Unit, His Majesty’s People’s Project, Thimphu, Bhutan

**Keywords:** Physiotherapy specialty, Physical and rehabilitation medicine, Holistic health, Human resources development, Evidence-based practice

## Abstract

Physiotherapy and rehabilitative services are an integral part of patient care, but in many developing countries they are not considered a priority and are either not available or not easily accessible to those who need them. Bhutan is one such country where healthcare is provided free of cost to all, but as of 2021 physiotherapy services were available only in 26 of 48 hospitals and 19 of 20 districts. The number of physiotherapy professionals per 10,000 population is 1.4 with significant rates of attrition. There is lack of awareness among patients and other health professionals about physiotherapy and rehabilitation services. The country needs to integrate physiotherapy and rehabilitation services into the overall health policy framework and develop proper planning of human resources and infrastructure to meet the current and future demands.

## Introduction

Physiotherapy services are integral component of holistic care of patients and are provided in different settings ranging from intensive care units to community settings [1]. Physiotherapists are part of a multidisciplinary team that provide preventive, curative, and rehabilitative care to promote healthy lifestyles, and provide therapy addressing musculoskeletal, neurological, cardiovascular and integumentary conditions [2, 3]. Rehabilitation aims to enhance physical, psychological, emotional, and social well-being for persons with disabilities by giving them opportunities for optimum participation and inclusion in society [2]. Physiotherapists also have increasing roles in acute and critical care units where appropriate therapies have shown to reduce the length of hospitalisation, prevent ventilator-associated pneumonia and physical impairments [4, 5]. Physiotherapy in appropriate settings also reduces the need for pharmacological agents for pain management such as opioids [6].

However, the role of physiotherapy in health systems is poorly understood in developing countries and patients do not have access to services [2]. Rehabilitation is often viewed as a fallback strategy when pharmacological interventions fail and receives minimal priority and resource allocation. For example in Sri Lanka, a country with good basic health indicators and a well-developed critical care system, physiotherapy services are available in only 91 of 100 state critical care units [7]. Though undergraduate training in physiotherapy is provided in Sri Lanka, there is a lack of regulated and systematic postgraduate training for physiotherapists [7]. Physiotherapy is often “linked” to persons with disabilities, which is only a small proportion of its clients [8]. The referral of patients to physiotherapists for assessment and management of musculoskeletal conditions is disproportionate to the number of affected individuals and is often delayed [9]. In South Africa, 90% of individuals with low back pain seen in primary care received pain medicine as their only form of treatment [10]. Likewise, in Ghana, the access to physiotherapy is limited due to out-of-pocket cost to the patients [11]. In Nigeria, clients had poor knowledge about physiotherapy as a form of treatment and patients did not seek physiotherapy services [12].

**Fig. 1:**
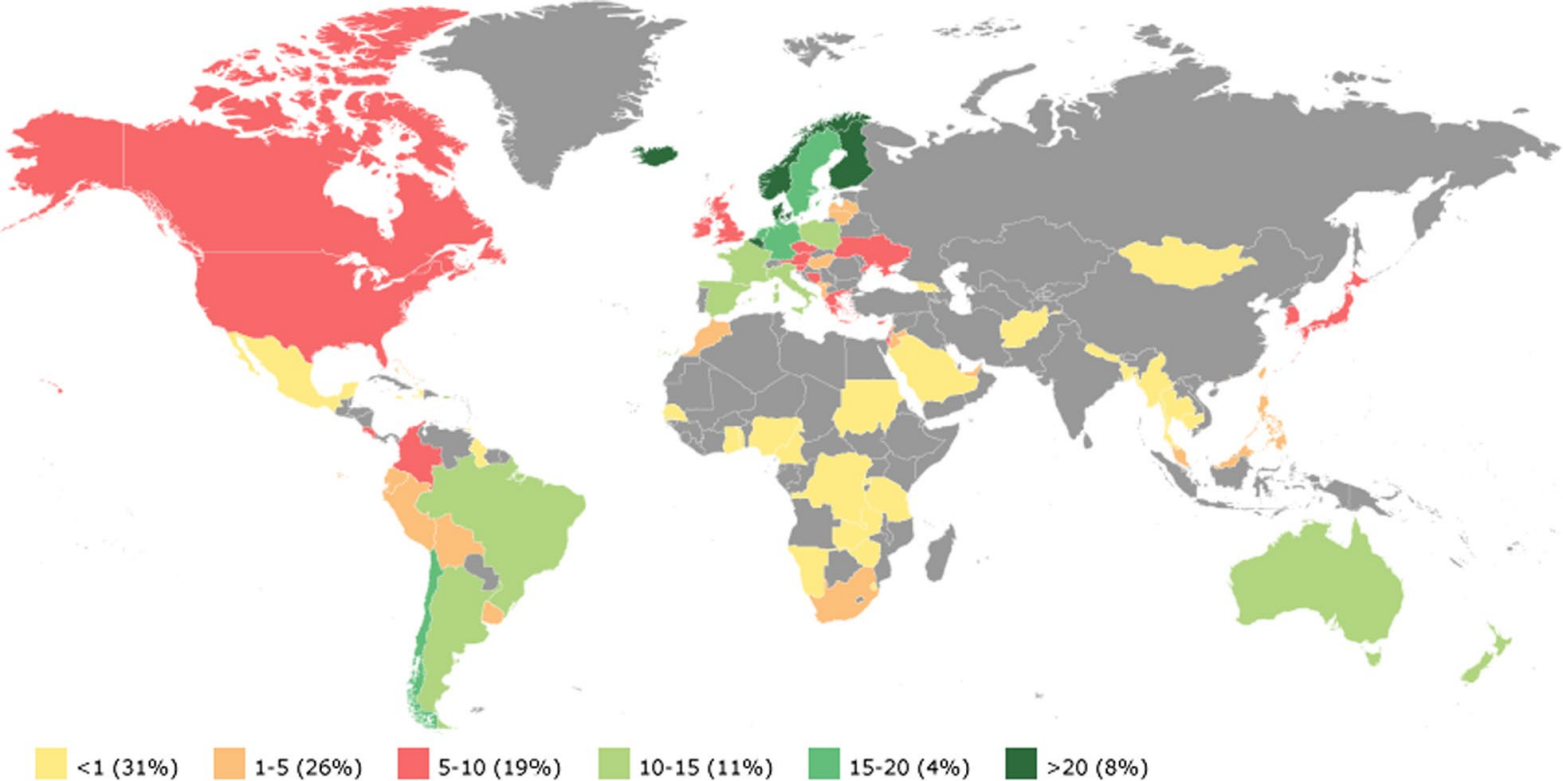
The availability of physiotherapists per 10,000 population among member countries of the World Physiotherapy  in 2018. Source: World Physiotherapy webpage (Accessed on 9 January, 2021)

Generally, human resources constraints in the health sector have been a chronic challenge in many developing countries with gross disparities across income levels. Among the member countries of the World Confederation for Physical Therapy (now World Physiotherapy) in 2018, developed countries had more physiotherapists per 10,000 population: > 20 in Finland, Iceland, Norway and Belgium; 10–15 in Australia, New Zealand, France, and Spain; 5–10 in the United States and Canada; and < 1 in developing countries such as Nepal, Bangladesh, Mexico, Burma, Thailand, and countries in Africia [13] (Fig. 1).

The situation of physiotherapy services in Bhutan is similar to that of many other developing countries. Bhutan is a small lower-middle income country with a population of 0.7 million people located in the Eastern Himalayas. It has a fully government-sponsored free health care system including physiotherapy services. However, amongst many other challenges faced by the health system in Bhutan, physiotherapy services have not yet been recognised as a priority sector and are yet to be integrated into all levels of patient care. This article aims to explore the challenges and opportunities faced by the physiotherapy professionals in Bhutan.

## Physiotherapy services in Bhutan

Physiotherapy services in Bhutan were established by an expatriate from Burma in 1984. As of 2021, the Royal Government of Bhutan trains physiotherapists in colleges in the neighbouring countries and offers diploma-level physiotherapy training at the Faculty of Nursing and Public Health, Khesar Gyalpo University of Medical Sciences of Bhutan, Thimphu [14]. The physiotherapy sector also receives support in the form of visiting experts from the Health Volunteer Overseas from the US and the Interplast team from Australia.

The physiotherapy department at the Jigme Dorji Wangchuck National Referral Hospital provides in-patient, out-patient, paediatric, hand, and maternal physiotherapy as well as assistive technology services [14]. The in-patient physiotherapy team provides services to the orthopaedic surgery, general surgery, medicine, and intensive care units facilitating early mobilisation and rehabilitation of patients.

In out-patient units, Women’s Health team provides antenatal and postnatal exercise programs. The Hand Therapy team works with patients following hand surgeries in addition to treating other musculoskeletal and neurological hand conditions, and providing splinting services. The musculoskeletal unit works with patients with non-traumatic musculoskeletal conditions such as low back pain, neck pain, osteoarthritis, etc. The neuro-rehabilitation team works with patients requiring long-term rehabilitation; and the paediatrics physiotherapy team works with children requiring physical rehabilitation. Similar in-patient and out-patient services are provided in two other Regional Referral Hospitals while only basic physiotherapy services are available in the district hospitals.

## Challenges for physiotherapy services in Bhutan

### Geographical inaccessibility

Bhutan is a mountainous country in the eastern Himalayas. Human settlement in the country is sparse and scattered over different geographical pockets. To reach medical and health services to the people of different communities, Primary Health Centres and Out-reach Clinics are established in the communities. As of 2021, Bhutan has 48 hospitals, 186 Primary Health Centres, and three Municipality Health Centres providing medical services to more than 95% of the population within three hours of travel distance [15]. However, physiotherapy services are available only in 26 hospitals and in 19 of the 20 districts in the country. Physiotherapy and rehabilitation services are provided over a long period of time, and most of the patients coming from villages to these select centres are unable to receive follow-up care due to distance and associated out-of-pocket cost related to travel [16]. This results in incomplete rehabilitation and readmission in the hospitals due to the development of secondary complications [16].

### Human resource constraints

**Fig. 2:**
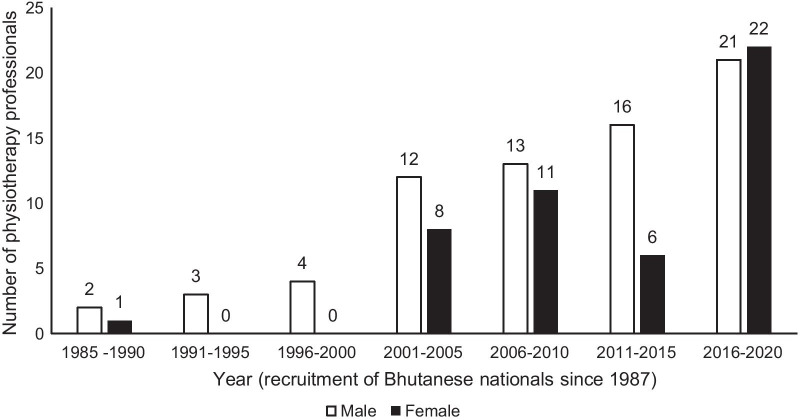
Recruitment of physiotherapy professionals into Civil Service in Bhutan between 1987 and 2020. Source:  Human Resource Division, Ministry of Health, Thimphu, 2021

As of 2021, there were 34 physiotherapists (5 on contract term of 2 years) and 68 physiotherapy technicians in the country with a small increase in the intake of professionals (Fig. 2). The ratio of physiotherapy professionals for every 10,000 population is 1.4 compared to 5.6 for doctors and 18.4 for nurses [15]. The Jigme Dorji Wangchuck National Referral Hospital caters to the largest patient load in the country and employs 14 physiotherapists and 21 physiotherapy technicians, the highest number of physiotherapy professionals among Bhutanese hospitals. The number of physiotherapy professionals available for populations in various districts in Bhutan is shown in Table 1.

**Table 1:** Physiotherapy professionals available per 10,000 population across 20 districts in Bhutan as of December, 2020

Dzongkhags	Population*	Physiotherapy professionals (*n*)	Physiotherapy professional per 10,000 population
Bumthang	17,820	2	1.12
Chhukha	68,966	5	0.72
Dagana	24,965	3	1.20
Gasa	3952	0	–
Haa	13,655	1	0.73
Lhuentse	14,437	2	1.38
Monggar	37,150	10	2.69
Paro	46,316	3	0.65
Pema Gatshel	23,632	2	0.85
Punakha	28,740	3	1.04
Samdrup Jongkhar	35,079	5	1.42
Samtse	62,590	4	0.64
Sarpang	46,004	8	1.74
Thimphu	138,736	41	2.96
Trashigang	45,518	4	0.88
Trashi Yangtse	17,300	1	0.58
Trongsa	19,960	1	0.50
Tsirang	22,376	2	0.89
Wangdue Phodrang	42,186	4	0.95
Zhemgang	17,763	1	0.56
Total	727,145	102	1.40

^*^Population and Housing Census of Bhutan 2017, National Statistics Bureau, Bhutan

With the increasing number of population requiring physiotherapy and rehabilitation services due to the increased life expectancy, road traffic accidents, occupational hazards, etc., the current human resource is inadequate in terms of numbers and adequately trained professionals. For example, Bhutan has only one specialist physiotherapist with specialisation in community-based rehabilitation. The present human resource training framework does not adequately recognise the importance of the expansion of services and improving the scope and quality of services provided [16]. In addition, specialisation in physiotherapy is under-recognised both by policy-makers and other health professionals.

Though physiotherapists have a path to upgrade their qualifications through higher education, due to the limitations of the present human resources framework, five physiotherapy technicians opted instead to pursue a bachelors degree in public health in 2020. In addition, attrition of physiotherapy professionals is a major problem. From 2019 to 2020, 11 physiotherapy professionals (3 physiotherapists and 8 physiotherapy technicians) have either resigned or went on to pursue other courses. There is no study on attrition of physiotherapy professionals in Bhutan but the study done among the physicians of Bhutan points out that improved pay, specialisation, improved conditions in the work places, and increase in distinct career paths including the non-clinical career pathways may improve job satisfaction and retention of health workers [17].

### Competency auditing

The World Health Organization and the International Labour Organization (ISCO code 2264) recognise physiotherapist as an autonomous professional [18]. However, the Bhutan Medical and Health Council (BMHC), a regulatory body for all health professionals in the country, categorises physiotherapists under the paramedics group that includes laboratory technicians, emergency medical responders, psychologists, clinical counsellors, nursing assistants, and public health field workers.

The recruitment of physiotherapy professionals into the civil service in Bhutan is done through a generic examination that does not adequately test clinical competencies such as communication and managerial skills, aptitude, commitment to patient-care and professional development. The BMHC lacks regulations that specify clinical competencies and skills of practicing physiotherapy professionals. Other than the requirement of Continued Medical Education credits for license renewal, the BMHC does not assess the clinical competencies of practising physiotherapy professionals.

### Infrastructure

The rehabilitation of patients with neurological conditions such as stroke, traumatic brain injury, and spinal cord injury requires adequate time, space and equipment. The World Physiotherapy standards require physical settings to have adequate space appropriate for the number and types of patients served, so as to ensure privacy, safety and comfort of the patients [19]. However, the physiotherapy department at the Jigme Dorji Wangchuck National Referral Hospital does not have adequate infrastructure. The Jigme Dorji Wangchuck National Referral Hospital in Thimphu and the Regional Referral Hospitals in Monggar and Gelegphu do not have separate beds in wards for rehabilitation resulting in premature discharge of patients and loss to follow-up [16]. The consequence is incomplete rehabilitation, readmission following secondary complications and additional costs for the healthcare system and the patients [16]. To address these issues, the government had proposed the establishment of a rehabilitation centre at Gidakom Hospital, Thimphu, through the Disability Prevention and Rehabilitation Program in the 11th Five Year Plan period 2013–2018 [16]. However, the proposal was not approved for the implementation [16].

### Misconceptions about physiotherapy care

There are many myths and misconceptions about physiotherapy among the public as well as health professionals. For example, the scope of physiotherapy is perceived as consisting of passive treatment modalities such as massage and electrophysical agents. Patients often opt for the surgeries instead of receiving physiotherapy services for musculoskeletal conditions with the belief that surgery is the treatment of pain. There are anecdotal evidence of patients perceiving physiotherapy treatments as painful experience of joint manipulations and unhelpful in regeneration of worn-out cartilage in conditions such as osteoarthritis. Such biomedical-based notions are a barrier to the effective use of physiotherapy management based on exercises and functional activities [20].

## Way forward

Bhutan is witnessing a rapidly changing demographic and socio-economic profile of the population. With increasing demand and expectation for better health and well-being, the country needs an overall strategic framework and policy to deliver to the present and future health needs. We suggest that one component of the framework should be dedicated to proper planning on the human resources for physiotherapy services with the scope of expansion of services, making it accessible to more people and specialisation of physiotherapists to provide better quality care. Attrition of physiotherapy professionals is aggravating the already inadequate human resources in the country. Therefore, innovative retention strategies need to be implemented [17].

The country needs a dedicated rehabilitation centre to cater to the specialised needs of patients requiring both short- and long-term rehabilitation services. There is a need to integrate physiotherapy services in patient care across all disciplines so that proper planning of care and follow-up can be made. Integration of physiotherapy services at the primary care level has a good scope in bringing about improvement of quality of life of older adults residing in villages and those who are unable to access hospitals.

There is a need to create awareness about the utility of physiotherapy and rehabilitation services through national television and print media, social media, inter-professional workshops, and maintenance of dedicated webpage to debunk common myths and provide evidence-based information.

## Conclusion

The demand for physiotherapy and rehabilitation services is on the rise in developing countries. However, many countries including Bhutan lack proper planning of human resources, infrastructure and policy framework to incorporate physiotherapy services into patient care at hospital and primary health care levels.

## Data Availability

Not applicable.
